# Adaptation of ACTivate Your Wellbeing, a Digital Health and Well-being Program for Young Persons: Co-design Approach

**DOI:** 10.2196/39913

**Published:** 2023-04-13

**Authors:** Menna Brown, Emily Lord, Ann John

**Affiliations:** 1 Swansea University Medical School Swansea University Swansea United Kingdom

**Keywords:** participatory design, well-being, health promotion, students, young persons, engagement, participation, web based, intervention, adherence, digital health, program, lifestyle changes, behavior changes, acceptance, mental health, physical health

## Abstract

**Background:**

ACTivate your wellbeing is a digital health and well-being program designed to support and encourage positive lifestyle behavior change. The website includes 5 lifestyle behavior change modules and a 12-week well-being intervention based on acceptance and commitment therapy. It was timely to adapt the resource for a new audience in the wake of the COVID-19 pandemic. Young persons’ mental health needs have increased substantially, and lifestyle behaviors play a critical role in both mental and physical health statuses.

**Objective:**

This study aimed to adapt an existing health and well-being website for use by young persons aged 16 to 24 years.

**Methods:**

A 3-staged participatory, co-design approach was adopted. The participants reviewed the existing program and provided feedback (stage 1) before cocreating new content (stage 2). Finally, the updated program underwent formative evaluation (stage 3). Two groups were created: one had access for 3 weeks and the other could self-select their study duration. The options were 3 weeks, 60 days, or 90 days. Outcome measures were the Warwick and Edinburgh Mental Well-being Scale, 4-item Patient Health Questionnaire, and Acceptance and Action Questionnaire version 2.

**Results:**

Stage 1 identified that the website was appealing to the new audience (19/24, 79%), and the 3 web-based focus group discussions explored data from the written review in more depth to identify and clarify the main areas for update and adaptation. Overall, 3 themes were developed, and the data informed the creation of 6 tasks for use in 5 web-based co-design workshops. Stage 2 led to the cocreation of 36 outputs, including a new name, new content, scenarios, images, and a new user dashboard, which included streaks and an updated color scheme. After the website update program was completed, 40 participants registered to use the website for formative evaluation (stage 3). Data analysis revealed differences in engagement, completion, and mean well-being after intervention between the 2 groups. The completion rate was 68% in the 3-week duration group, and well-being scores improved after intervention.

**Conclusions:**

Young persons engaged actively with the participatory design process. The participants discussed the updates they desired during the web-based discussions, which worked well via Zoom (Zoom Video Communications Inc) when small groups were used. The participants easily cocreated new content during the web-based co-design workshops. The web-based format enabled a range of participants to take part, share their ideas, search for images, and design digital content creatively together. The Zoom software enabled screen sharing and collaborative whiteboard use, which helped the cocreation process. The formative evaluation suggested that younger users who engage more with the website for a shorter duration may benefit more.

## Introduction

### Background

The Well-being of Future Generations (Wales) Act [1] placed a legal responsibility on Welsh public services to improve the “economic, social, environmental and cultural well-being of its area.” Prosperity for All [2] continued to build on the Welsh Assembly Government’s commitment to health and well-being. This momentum has continued with the publication of “Health and Social Care in Wales - COVID-19, Looking forward” [3], as the detrimental impact of the COVID-19 pandemic on the population’s mental health continues to emerge [4-9]. In addition, research suggests that lifestyle behaviors were negatively impacted by the pandemic [10-13]. Lifestyle behaviors have an indirect impact on mental and physical health and are leading causes of noncommunicable diseases, a public health issue of global concern [14].

ACTivate your wellbeing is a digital health and well-being program previously created [15] to support and encourage public sector staff to improve their lifestyle behavior and boost their well-being. It was co-designed with anticipated end users using a participatory design (PD) approach [16]. It was timely to adapt the resource for a new audience in the wake of the COVID-19 pandemic. The mental health of students and young persons has been declining [17,18]. Concurrent with the immediate impact of the pandemic, substantial deterioration in the well-being and anxiety of undergraduate students have been reported [19-21].

In the United Kingdom, the National Student Union highlighted that 52% of students perceived their mental health to be negatively affected by the pandemic [22]. Chen and Lucok [23] surveyed 1173 UK students and found that 50% had high levels of anxiety and depression, above the clinical cutoff points, and that female students fared worse. The situation is worsened by a decline in the available services [24] and an increase in demand [25]. A survey reported that 60% of American students felt that the pandemic made it more difficult to access mental health care [26].

The 2021 National Health Service digital survey of children and young persons in England reported an increase in the rate of probable mental disorders among people aged 17 to 23 years; 52.5% experienced deterioration [27]. The mental disorders highlighted for this age group included eating disorders, sleep problems, loneliness, and substance abuse. In addition, this age group reported lower social and family connectedness and family functioning than their counterpart in the 2017 survey [27]. This is in line with longitudinal data that highlighted the direct impact of COVID-19–related stressors, particularly social or relational stressors, on young persons’ mental health, well-being, and life satisfaction [28]. Specifically, young persons who experienced greater COVID-19–related stressors in 2020 reported more anxiety and depression and lower satisfaction with life since the pandemic [28]. This has implications for young persons as they recover and move forward from the past year’s disruption.

Furthermore, the lifestyle behaviors of students and young persons were affected. For example, recent findings from an extensive literature review found a reduced rate of physical activity, an increased rate of obesity and poor eating habits, and micronutrient deficiencies related to unhealthy diets in children and adolescents [29]. Similar patterns were observed in students [30,31].

Prior research has shown that poor lifestyle behaviors are likely to cluster with multiple unhealthy behaviors [32]. Physical health and mental health have a bidirectional relationship; therefore, worsening of one has implications for the other. Freely available, digital resources are needed to support young persons’ health and realize improvements in their well-being [33].

ACTivate your wellbeing [15] houses 5 lifestyle behavior change modules: quit smoking, regular exercise, eat healthily, weight optimization, and alcohol reduction. Each module includes interactive content that promotes health and provides motivational information based on 4 health promotion theories—the health belief model [34]; theory of planned behavior [35]; plan, do, study, act [36]; and self-regulatory model [37]—and an interactive 12-week well-being intervention based on acceptance and commitment therapy (ACT). ACT is considered a third-wave cognitive behavioral therapy and is philosophically rooted in functional contextualism [38,39] and relational frame theory [40]. ACT interventions include mindful exercises that promote contact with the present moment. Earlier systematic reviews and meta-analyses have found web-delivered ACT to be effective for the management of depression and anxiety [41], and others [42-46] report its effectiveness in both group and individual settings.

### Goal of This Study

Thus, this study aimed to adapt ACTivate your wellbeing to a new audience by following a co-design approach [47]. Co-design and participatory approaches are widely advocated in the design and development of web-based interventions as a collaborative inclusive design approach that can result in increased engagement [47-49] and limit the likelihood that the end “product” will be rejected. The active and collaborative design approach is valued for its ability to offer diverse users a voice in the design process. The emphasis on respect and equality enables a shift in focus away from the consideration of purely technical requirements toward an understanding of end users’ needs.

Specifically, the involvement of anticipated end users, similar to the involvement of expert patients in psychological studies, can highlight, early on, key information regarding users’ needs; can facilitate a deeper understanding of users’ knowledge and values [50]; and have been increasingly politically and socially advocated in health care [51]. However, participation does not guarantee the uptake of the intervention by end users [52].

Specific objectives of this study were as follows:

1. Consult and engage with young persons to explore their views on the existing website and understand its perceived usefulness
2. Consult and engage with young persons to identify areas of the existing website that need to be updated and adapted to suit them
3. Consult and engage with young persons to explore ways to encourage sustained engagement with the website
4. Collaborate with young persons to co-design new content representative of their experiences with a specific focus on the well-being intervention
5. Collaborate with young persons to co-design the layout, wording, and design features, including imagery, to be used throughout the website to ensure that they meet their preferences
6. Update the website using the co-designed content
7. Pilot the updated website to explore engagement and gather additional feedback

## Methods

### Ethics Approval

Ethics approval was granted by the Research Ethics Committee of the Swansea University Medical School (2020-0010A 16.6.20).

### Participants

The participants were young persons aged 16 to 24 years currently engaged in academic studies in Wales, United Kingdom.

### Inclusion Criteria

The inclusion criteria used in this study were as follows:

1. A young person enrolled in further or higher education aged 16 to 24 years
2. Ability to provide informed consent
3. Access to an internet-enabled device

### Recruitment

Digital study flyers and advertisements were posted across one faculty virtual learning environment within a UK University and shared locally via social media platforms created for the study. Interested young persons were required to email the researcher (EL), and then they were sent further information, including a written consent form and participant information sheet. Vouchers worth between £5 (US $6.5) and £20 (US $26) were offered as incentives for participation in different stages of the study.

### Procedure

A 3-staged design process was followed, which included rapid prototyping, reflection, collective review, and rereview and cocreation of new content [53].

#### Procedure Stage 1: Initial Exploration

The participants were provided with access to the study website via a dummy account and asked to review the existing site and resources by completing a structured feedback form that asked about initial impressions and perceived usefulness and for general comments. Then, it asked for specific feedback on each section of the website. This generated initial insights into the new audience’s opinions. Responses were collated and summarized to inform web-based focus group (FG) discussions, which were held to facilitate greater exploration of the participants’ opinions and thoughts on how to change and adapt the website overall and well-being intervention specifically. As such, the participants were asked to discuss each section of the website identified in the review stage as in need of update and adaptation to the new audience. These included the home page, user dashboard, profile area, 5 different lifestyle modules, and well-being intervention. In addition, the participants were asked to consider design elements, the use of gamification features, and ways to enhance engagement and adherence. The principal investigator facilitated the FGs and used visual aids, including website images and resources, to stimulate discussion. All FGs were audio and video recorded using the Zoom software (Zoom Video Communications Inc).

#### Procedure Stage 2: Co-design Workshops

The participants provided written consent before taking part in the web-based co-design workshops. A range of times and dates were offered to ensure that interested participants could attend at a convenient time that fitted around their studies, which at the time was held on the web owing to the COVID-19 pandemic. The participants were welcomed, and the purpose of the workshops was outlined.

Informed by the first stage, 6 tasks were created for use in the web-based workshops (Textbox 1). The participants completed 2 tasks in each 2-hour workshop, and a short break was included in the middle. EL facilitated the workshops. The participants were provided with a group resource card and guidance on how to complete the task at the beginning of each workshop before being asked to work collaboratively to cocreate the changes they wanted to see on the website. The participants had previously taken part in the FG discussions, so they had met on the web once already. Introductions were not made, as screen names were included on Zoom. Facilitators did not take part in the participant discussions. A minimum of 3 participants were included in each workshop. The participants were given space and time to talk through the tasks and consider what they wanted to focus on. This ensured that the adaptations were created by the participants, and all could take part. After the completion of each task, the facilitator asked the participants to discuss the output and ideas they had created. This allowed the participants an opportunity to summarize, share, and review their outputs.

Successive workshops followed the same format but had the added component of a short review of the previous workshop’s outputs at the beginning. For example, in workshop 2, participants were shown the updated home page that they had created in workshop 1. They had the opportunity to review the changes and add their suggestions. This process facilitated rapid prototyping.

After the completion of the workshops, the researchers reviewed the outputs, updated the website with the new images and text, and commissioned the structural updates to be implemented.

**Co-design workshop tasks.**

Workshop 1

- Task 1a: redesign the home page such that it suits your and your peers’ style and layout preferences
- Task 2a: develop 2 scenario or examples to be included in the well-being intervention

Workshop 2

- Task 1b: redesign the user dashboard that it suits your and your peers’ ideas and suggestions for encouraging engagement
- Task 2b: update the well-being intervention’s structure and interaction points such that they suit your needs and encourage engagement

Workshop 3

- Task 1c: redesign the track your progress feature such that it suits your and your peers’ ideas and suggestions for encouraging engagement
- Task 2c: update the well-being intervention and make the changes you would like to see

Workshop 4

- Task 1a: redesign the home page such that it suits your and your peers’ style and layout preferences
- Task 2a: develop 2 scenario or examples to be included in the well-being intervention

Workshop 5

- Task1d: redesign the user profile area such that it suits your and your peers’ style and layout preferences
- Task 2b: update the well-being intervention’s structure and interaction points such that they suit your needs and encourage engagement

#### Procedure Stage 3: Formative Evaluation

Participants from stages 1 and 2 (and additional anticipated end users) were invited to visit the updated website and register as a user (the registration process is detailed elsewhere [16]). They were required to enroll in at least 1 lifestyle module and the well-being module. Half of the users (20/40, 50%) were given a 3-week study duration, and half (20/40, 50%) were offered the opportunity to self-select their desired duration from several options: 3 weeks, 60 days, or 90 days. This distinction was undertaken during registration based on their “university degree” (identified during registration). The external web programmer was asked to create 2 user profiles; this meant that when a user identified themselves as a “medical student,” they were shown the available duration options. All choices were available to medical students but other types of students or users were only given the 3-week study duration option. This was not randomized; medical students were offered the opportunity to self-select based on their preferences identified in stage 2.

The participants were encouraged to use the website frequently. Email reminders were sent to those who opted in. The well-being module included daily activities to complete, mindful mediations, ACT skills to learn, experiential exercises, and a visualization or metaphor. Each lifestyle module included a weekly progress monitoring area (displayed via the user dashboard) and goal-setting tool. At the end, the participants were asked to complete the same outcome measures completed at registration and to provide feedback via an embedded survey. Data were extracted from the website and analyzed after the 12-week study period. The participants who completed all the requirements were entered into a prize draw to win a voucher worth £50 (US $65).

### Outcome Measures

The 14-item Warwick Edinburgh Mental Well-Being Scale (WEMWBS) is a validated measure of mental well-being in the general population and is responsive to change at both the individual and group levels. The validated measure enables researchers to establish where a specific population falls in relation to published national population averages following the use of interventions of 2-week (or longer) duration [52-55]. The 5-point Likert scale measure (which asks for responses between “none of the time” and “all of the time”) includes questions relating to both eudaimonic (ie, positive functioning) and hedonic (ie, life satisfaction) perspectives of subjective well-being [54], and only positively worded items are used. The questions cover psychological functioning, cognitive evaluation, and emotional aspects of subjective well-being. The classification of the WEMWBS is presented as a mean. A score of ≤43.5 is considered a screening threshold for depression [52].

The Patient Health Questionnaire-4 (PHQ-4) with 4 items is an ultrabrief self-report questionnaire [56]. The internal consistency is considered excellent (Cronbach α=.78), with adequate construct validity correlations with the Rosenberg Self-Esteem Scale, questionnaire on life satisfaction, and resilience scale (*r*=−0.49 to 0.40, *r*=0.39 to −0.39, and *r*=0.35 to 0.28, respectively) [56].

The 7-item Acceptance and Action Questionnaire version 2 (AAQ-II) is a validated, 1-factor measure of psychological inflexibility [57,58]. Psychological flexibility refers to “the ability to fully contact the present moment in order to engage behavioural patterns supporting movement towards valued end*”* [59]. Acceptance is an example of psychological flexibility, and experiential avoidance is an example of inflexibility [57]. Higher levels of psychological inflexibility indicate greater psychological distress. The cutoff points have not been published. However, the authors suggested that scores between 24 and 28 indicate depression or anxiety [57].

### Data Analysis

#### Data Analysis Stage 1

Qualitative data were analyzed using the 5-stage inductive thematic analysis process advocated by Braun and Clarke [60]. First, the principal investigator refamiliarized herself with the data by reading and rereading the FG transcripts. Second, the transcripts were annotated, and initial codes were generated using a line-by-line approach, summarizing the data to capture the essence of the participants’ thoughts and views. The codes were compiled in a formal coding document with the identified example extracts. Stage 3 involved searching for themes across the data set using the coding structure. The questions asked during the discussions and structure of the discussions guided the theme development, which focused on identifying areas for adaptation highlighted by the participants. The developed themes [61] were discussed (EL) to support triangulation of interpretation. Stage 4 involved the review and refinement of the themes, and stage 5 involved the development of theme names.

#### Data Analysis Stage 2

Coproduced designs, images, and resources were created during the workshops, discussed by the participants, and shared with the facilitator. The outputs were directly added to the website.

#### Data Analysis Stage 3

Recruitment was measured based on the number of registered users.

Engagement was measured based on module enrollment, use of interactive features (ie, “try now” and “track your progress” located within the well-being intervention area and lifestyle modules, respectively), user points (which were automatically generated by the system and displayed to the users via the user dashboard; participants who earned 20 to 40 points were considered high users, whereas those who scored 0 to 19 points were considered low users), review of google analytics embedded in the website, (average “log-on rate”) and module completion. Adherence was measured based on the completion of the 3 outcome measures at baseline and after intervention, calculated as a percentage. Finally, satisfaction and usefulness were assessed using the embedded feedback form.

## Results

Results are presented per stage. Participant characteristics are presented in Table 1.

**Table 1:** Participant characteristics (N=81).

Stage and task			Duration (minutes)		Participant, n (%)		Female participant, n (%)		Year of study: participant, n (%)
**Stage 1 (n=24)**									
	Website review	N/A^a^		24 (100)		14 (58)		Year 13: 1 (4) Year 2: 1 (4) Year 3: 10 (42) GEM1^b^: 1 (4) GEM2: 7 (30) GEM3: 3 (12) GEM4: 1 (4)	
	Focus group 1	90		5 (21)		5 (100)		GEM2: 4 (80) GEM3: 1 (20)	
	Focus group 2	79		5 (21)		3 (60)		GEM1: 1 (20) GEM2: 1 (20) GEM3: 2 (40) GEM4: 1 (20)	
	Focus group 3	65		2 (8)		2 (100)		GEM2: 2 (100)	
**Stage 2 (n=17)**									
	Co-design workshop 1	120		3 (18)		3 (100)		GEM2: 3 (100)	
	Co-design workshop 2	120		4 (24)		4 (100)		GEM2: 4 (100)	
	Co-design workshop 3	120		3 (18)		3 (100)		GEM2: 3 (100)	
	Co-design workshop 4	61		3 (18)		1 (33)		Year 2: 1 (33) Year 3: 2 (67)	
	Co-design workshop 5	90		4 (24)		2 (50)		Year 2: 2 (50) Year 3: 2 (50)	
**Stage 3 (n=40)**									
	Pilot review	3 to 12 weeks		20 (50)		16 (80)		GEM1: 2 (10) GEM2: 10 (50) GEM3: 6 (30) GEM4: 2 (10)	
	Pilot review	3 weeks		20 (50)		11 (55)		Year 13: 5 (25) Year 2: 12 (60) Year 3: 3 (15)	

^a^N/A: not applicable.

^b^GEM: graduate entry medical student.

### Results of Stage 1

#### Overview

A total of 24 participants reviewed the website. The feedback identified that each area of the website could be adapted to suit the new audience. The feedback form asked the participants whether they considered the study website to be appealing. Most participants agreed (19/24, 79%). Areas for adaptation and update were collated section wise. Each area had suggestions for change.

Then, 12 participants took part in 1 of the 3 web-based FG discussions. Thematic analysis enabled the researcher to develop 3 themes [61], which focused on the perceived usefulness of the website, areas for adaptation, and the issue of engagement with and adherence to web-based health interventions [41]. As such, the themes were “programme relevance,” “areas for adaptation and update,” and “engagement and adherence.” These are outlined in subsequent sections.

#### Results of Stage 1: Theme 1—Program Relevance

The data indicated that the participants considered the website to be both relevant and useful. In particular, the participants highlighted their appreciation for and interest in the well-being intervention, but they also noted the benefits of having access to a multifaceted program. However, the participants wanted the program to be available via an app rather than a website. The following extracts demonstrate this:

I think the activate your well-being, was a really good resource, especially for us on our course.

GEM2 female, FG1

I think it would be a good source for, like, especially a first year is because they can learn these techniques for when they really need them.

GEM2 female, FG1

...if it was possible to be in an app form rather than a website form because I think in this way you can access it a lot easier. I can access it pretty much more times of day when you work or lectures or whatever. It’s just easier

GEM4 female, FG2

#### Results of Stage 1: Theme 2—Areas for Adaptation and Update

The identified areas for adaptation are organized per website section. This section focuses on the adaptations identified for the well-being module. The participants called for a more condensed and streamlined resource, particularly for young persons who had not previously used a well-being intervention or who had multiple demands on their time (eg, studying and living away from home for the first time):

When looking at each week for activate your well-being, the fact that there was a lot of information. A lot of resources and it was really great. But for someone who’s never like done anything with wellbeing before I think it could become overwhelming

GEM2 female, FG1

Specific topics that would be of interest and relevance to the young persons, including “exam stress,” “how to talk to patients,” “what to do on a Sunday,” “things I wish I’d known,” “how to cope with difficult clinical experiences,” and “how to support a peer,” were suggested for inclusion in the intervention.

The use of images and design features was also discussed, and the participants considered it important to include male and female images within the module to highlight that well-being is not a female topic. This is evidenced by the following extract:

Keeping it as a male is important because like looking after your emotions is often something that men tend to be discouraged from so it’s subliminally may be encouraging men to take the module

GEM2 female, FG1

#### Results of Stage 1: Theme 3—Engagement and Adherence

When asked how to encourage engagement with and continued use of the website for a duration of ≥3 weeks, the participants made several suggestions for the website overall. These included making changes to the user dashboard so that it included “streaks” as a way to encourage users to log on each day. This is similar to existing apps such as Duolingo that the participants were familiar with. In addition, the use of a prize draw was suggested. This was later adopted in stage 3:

You could use there is the idea of a streak. So things like you know rewarding you for coming back and coming back. And then if you don’t come back. Or you lose it there is that risk

GEM3 male, FG2

I think I would be much more engaged. If there was something in it you know, a potential prize maybe

GEM1 male, FG2

Using the data from both components of this stage, a list of changes was identified for immediate update. These are listed in Table 2. However, not all suggestions could be implemented owing to the time and cost required to program the changes, or the structure of the existing website could not accommodate the changes. This is noted in the *Discussion* section and considered a limitation of this study.

Finally, the findings from stage 1 were used collectively to inform the development of 6 tasks, which were used in stage 2. Two types of tasks were decided on: (1) design-focused tasks (1a-1c in Multimedia Appendix 1) and (2) tasks that focused on developing and updating the well-being intervention (2a-2c in Multimedia Appendix 1). These reflected the participants’ primary interests and were considerate of the time frame available. These tasks are presented in Textbox 1. The resource card for task 1a is presented in Multimedia Appendix 1 as an example.

**Table 2:** Updates and adaptations identified in stage 1.

Change number	Changes implemented	Location of change
1	The program name was updated from “Champions for Health” to “ACTivate your wellbeing”	Home page
2	The user dashboard link was added to the main menu on the home page to allow direct access	Home page
3	User dashboard functionality, design, and layout were updated to encourage engagement via the use of streaks	User dashboard
4	Modules that the participants had not enrolled in were removed from the display area, ensuring personalization	User dashboard
5	The profile page was updated to allow users to upload a personal profile picture and to tailor the information displayed to their tastes via added functionality	Profile page
6	The registration page was formatted for specific student courses with additional drop-down menu options	Registration page
7	The BMI tracker was adjusted for improved accuracy	Weight optimization module
10	Student-focused recipes and budget friendly ideas were prepared	Weight optimization module
13	Additional guided mindful meditations were added to the well-being intervention, tailored scenarios for students and young persons were added, and additional resources and links were included for mental health support	Well-being intervention
15	Additional resources and links were included for mental health support	Well-being intervention

### Results of Stage 2

A total of 17 participants took part in 1 of the 5 web-based workshops, and the mean duration was 102 (SD 24.19711; range 61-120) minutes. The workshops produced 36 outputs, described in Table 3, all of which were implemented.

**Table 3:** A list of all workshop outputs.

Output number	Description of output	Reference figure or textbox
1	Agreement of new name	Figure 1
2	New icon for weight optimization	Figure 2
3	New icon for quit smoking	Figure 2
4	New icon for regular exercise	Figure 2
5	New icon for drink responsibly	Figure 2
6	New icon for eat healthily	Figure 2
7	New icon for well-being intervention	Figure 2
8	New blurb to describe and encourage enrollment in the weight optimization module on the home page	Figure 3
9	New blurb to describe and encourage enrollment in the quit smoking module on the home page	Figure 3
10	New blurb to describe and encourage enrollment in the regular exercise module on the home page	Figure 3
11	New blurb to describe and encourage enrollment in the drink responsibly module on the home page	Figure 3
12	New blurb to describe and encourage enrollment in the eat healthily module on the home page	Figure 3
13	New blurb to describe and encourage enrollment in the well-being intervention on the home page	Figure 3
14	New blurb to encourage registration to the website, located under the name	Figure 3
15	New color scheme for each lifestyle module and the well-being intervention	Figure 4
16	New layout for the user dashboard to include “streaks” to encourage engagement, including description of how the streak should be operationalized	Figure 4
17	Additional content for the well-being intervention—example of MIND resources, which the participants found useful	N/A^a^
18	New images for track your progress	Figure 5
19	New layout and wording for the goal-setting area	Figure 6
20	New placement of the well-being intervention on the home page. It was to be moved to the first option as opposed to the last option	N/A
21	New scenario to be used in week 2 of the well-being intervention	N/A
22	New scenario to be used in week 3 of the well-being intervention	Textbox 2
23	New scenario to be used in week 4 of the well-being intervention	N/A
24	New scenario to be used in week 5 of the well-being intervention	N/A
25	New scenario to be used in week 6 of the well-being intervention	N/A
26	New scenario to be used in week 7 of the well-being intervention	Textbox 2
27	New titles for the subsections in the well-being intervention, displayed initially in week 1 and used throughout to organize content	N/A
28	Inclusion of 4 more guided mindful mediations to ensure that all 12 weeks of the intervention included this option; previously, only 8 weeks included this	N/A
29	Addition of more testimonials to the home page	N/A
30	New color needed for hyperlinks	N/A
31	Rewording of the 3-step banner on the home page to make it clearer that there was a choice of duration available	N/A
32	Profile page layout alterations, including the addition of the option to upload a profile picture	N/A
33	New image and updated example for week 9	N/A
34	New image for week 1 of the well-being intervention depicting a set of headphones	N/A
35	New image for use in week 6 of the well-being module week 6	N/A
36	Going home checklist	N/A

^a^N/A: not applicable.

**Figure 1:**
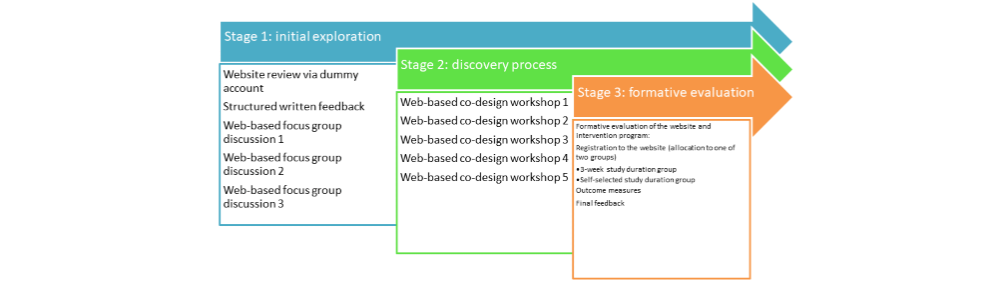
Study diagram.

**Figure 2:**
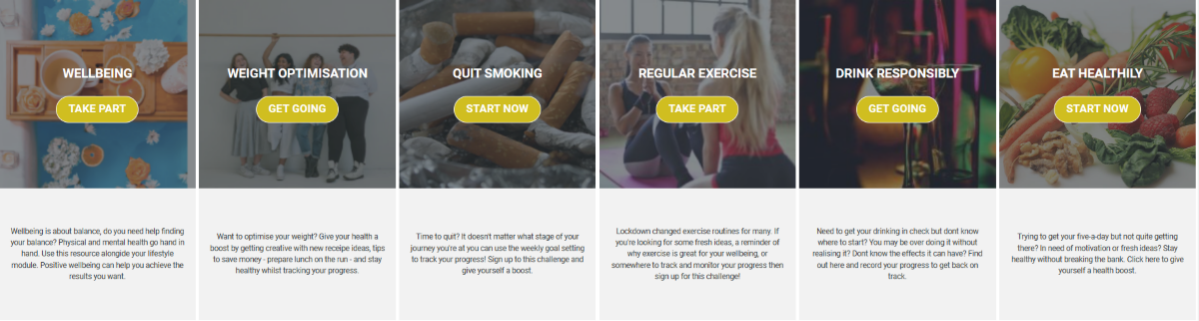
Updated images and module descriptions.

**Figure 3:**
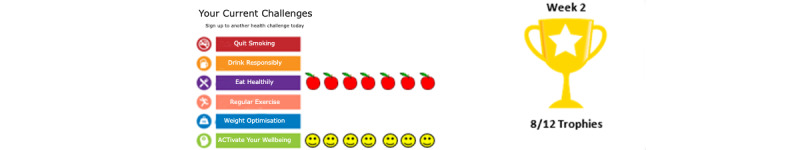
Updated user dashboard.

**Figure 4:**
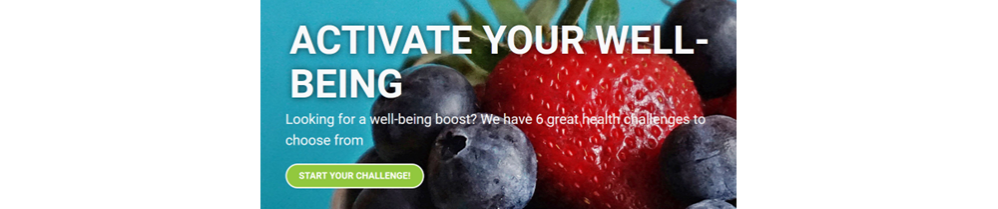
Updated home page text.

**Figure 5:**
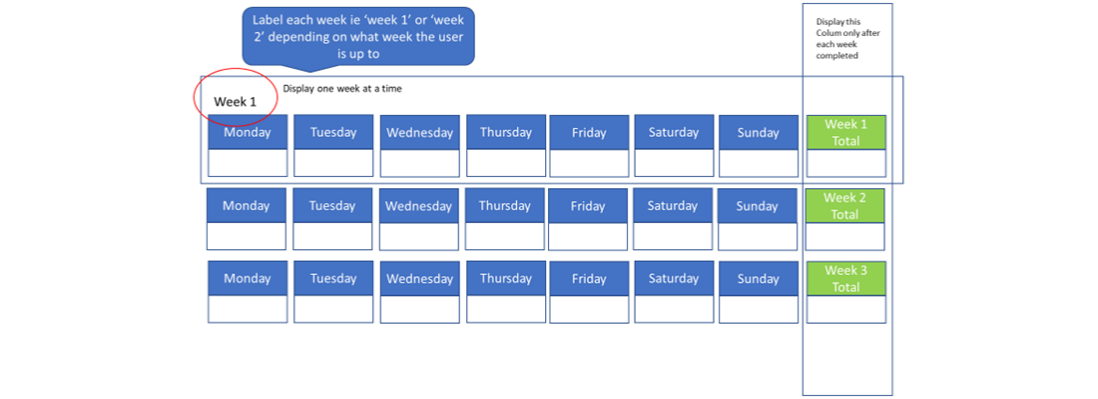
Updated track your progress display.

**Figure 6:**
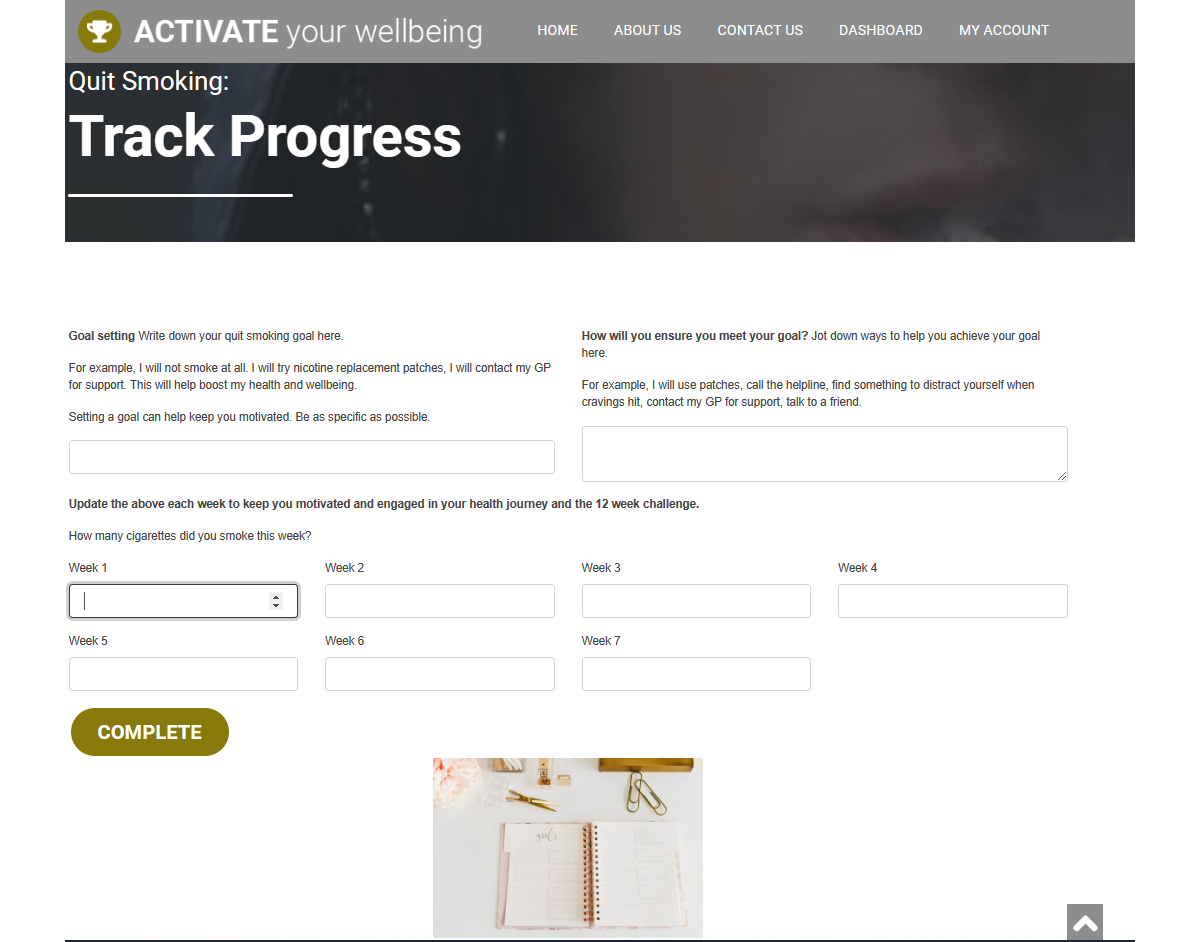
Updated text and display.

**Example developed for the well-being module.**

Week 3

- Andrew [not real name] was never a big drinker before, however he worried he wouldn’t make friends or fit in if he didn’t attend the nights out. But he has just found himself spending more time hungover and not making any valuable friendships and is now feeling stressed that he is behind on studies. Completing week 3 has made him re-evaluate his values and alternative ways that he could make friendships. Next week he is planning on reaching out and joining a sports team instead.

Week 7

- Tom [not real name] is on a LOCS with a gastro consultant. He has spent time studying in advance but when questions are directed at him, he doubts his ability and freezes up. He has got to the point where he would rather stay silent than embarrass himself with the wrong answer. He is now worried for upcoming placements where he maybe in a similar situation.

But after taking part in week 7, he now realises the value in trying. Even if he is wrong, he is more likely to learn and not miss out on valuable teaching opportunities.

### Results of Stage 3

#### Overview

A total of 40 participants took part in the formative evaluation. The majority (28/40, 70%) opted for email reminders. Of the 20 participants who could self-select their study duration, 2 (10%) selected the 21-day challenge, 10 (50%) selected the 60-day challenge, and 8 (40%) selected the 90-day challenge. The remaining participants were automatically enrolled for the 3-week (21-day) challenge.

#### Outcome Measures

Table 4 presents the 3 preintervention and postintervention outcome measures for the 40 participants per group. No statistically significant changes were observed.

At baseline, the group that self-selected their study duration (n=20) had a mean WEMWBS score of 45.3 (SD 6.752; 20/20, 100%), and the combined PHQ-4 mean score was 2.25 (SD 1.552; 20/20, 100%). Both were below the reported cutoff points for possible common mental disorder (CMD). At baseline, the mean AAQ-II score was 18.35 (SD 5.669; 20/20, 100%), which suggested positive psychological flexibility and was also below the cutoff point for symptoms of CMD.

Only 5% (1/20) of participants completed the outcome measures after the intervention. Thus, no meaningful comparison could be drawn between the preintervention and postintervention results.

At baseline, the 3-week study duration group (n=20) had a mean WEMWBS score of 45.4 (SD 6.50; 20/20, 100%); this increased after the intervention to 49.07 (SD=6.68, 15/20, 75%). The difference neared statistical significance (paired samples 2-tailed *t* test; *P*=.05). The mean PHQ-4 score at baseline was 3.20 (SD 2.46; 20/20, 100%), and the mean PHQ-4 score after the intervention was 2.15 (SD 1.46; 15/20, 65%). A paired samples 2-tailed *t* test did not detect a significant difference (*P*>.99). The mean AAQ-II score at baseline was 18.55 (SD 8.98; 20/20, 100%), and the mean AAQ-II score after the intervention was 19.77 (SD 9.48; 15/20, 65%). A paired samples 2-tailed *t* test did not detect a significant difference (*P*=.44).

**Table 4:** Preintervention and postintervention outcome measures.

Group and outcome measures			Value, n (%)		Before intervention, mean (SD)		Value, n (%)		After intervention, mean (SD)
**Self-selected study duration (n=20)**									
	WEMWBS^a^	20 (100)		45.3 (6.752)		1 (5)		51 (0)	
	PHQ-4^b^	20 (100)		2.25 (1.552)		1 (5)		0 (0)	
	AAQ-II^c^	20 (100)		18.35 (5.669)		1 (5)		13 (0)	
**3-week study duration (n=20)**									
	WEMWBS	20 (100)		45.45 (6.501)		15 (75)		49.07 (6.681)	
	PHQ-4	20 (100)		3.20 (2.462)		15 (75)		2.07 (1.387)	
	AAQ-II	20 (100)		18.55 (8.988)		15 (75)		19 (9.047)	

^a^WEMWBS: Warwick Edinburgh Mental Well-Being Scale.

^b^PHQ-4: Patient Health Questionnaire-4.

^c^AAQ-II: Acceptance and Action Questionnaire version 2.

#### Module Enrollment, Engagement, and Completion

Of the 20 participants in the self-selected study duration group, 5 (25%) did not enroll in any modules, 4 (20%) enrolled in 1 module, approximately half (n=8, 40%) enrolled in 2 modules, 2 (10%) enrolled in 3 modules, and 1 (5%) enrolled in 4 modules.

Module engagement varied (Table 5). The average engagement time was 10 hours and 56 minutes. The home page received the most views, followed by the dashboard and well-being home page.

In the self-selected study duration group, none of the participants completed their self-selected challenge duration; however, in the 3-week study duration group, a 13% completion rate was observed. Those who did not complete reported “lack of time.”

In the 3-week study duration group (n=20), a total of 4 (20%) participants did not enroll in any lifestyle modules, 1 (5%) enrolled in 1 lifestyle module, 4 (20%) enrolled in 2 lifestyle modules, and 1 (5%) enrolled in 4 lifestyle modules.

A total of 10 (20%) participants were classified as high engagers. The mean engagement for high engagers was 25.3 user points, and the mean engagement for low engagers was 4.7 points. When the user points were explored, a difference among the postintervention WEMWBS scores was observed. Those classified as high engagers had a higher mean WEMWBS score after the intervention (mean 52.63, SD 6.368; 8/10, 40%) than those classified as low engagers (mean 45, SD 4.546; 7/10, 35%). This was statistically significant (*P*=.02) in an independent samples 2-tailed *t* test.

Most participants (n=14/20, 70%) completed their selected modules (Table 6). Drink responsibly had a 100% (3/3) completion rate, well-being had a 78% (14/18) completion rate, weight optimization had a 60% (3/5) completion rate, regular exercise had a 55% (5/9) completion rate, and eat healthily had a 50% (3/6) completion rate.

User points were also used to determine engagement.

**Table 5:** Self-selected study duration group (n=20) enrollments, engagements, and module completions.

Module	Enrollment, n (%)	Engagement, n (%)	Completion, n (%)
Quit smoking	0 (0)	0 (0)	0 (0)
Alcohol reduction	2 (10)	1 (5)	0 (0)
Weight optimization	4 (20)	3 (15)	1 (5)
Eat healthily	3 (15)	1 (5)	1 (5)
Regular exercise	8 (40)	5 (25)	1 (5)
Well-being	13 (65)	7 (35)	1 (5)

**Table 6:** Self-selected study duration (n=20) group enrollments, engagements, and module completions.

Module	Enrollment, n (%)	Engagement, n (%)	Completion, n (%)
Quit smoking	0 (0)	0 (0)	0 (0)
Alcohol reduction	3 (15)	3 (15)	3 (15)
Weight optimization	5 (25)	5 (25)	3 (15)
Eat healthily	6 (30)	6 (30)	3 (15)
Regular exercise	9 (45)	9 (45)	5 (25)
Well-being	18 (90)	18 (90)	14 (70)

#### Health Outcomes

Health improvements over the 3-week duration were recorded only for those in the 3-week study duration. These included the following: there was a reduction in alcohol consumption from a mean of 19.33 units to 8 units, which was not significant (*P*=.20); there was an increase in the consumption of fruits and vegetables from a mean of 12 portions per week at baseline to 17 portions per week after 3 weeks, which was not significant (*P*=.29); and half of the participants who reported that they wanted to gain weight were successful, as was the 1 person who selected to maintain their weight.

#### Formative Evaluation Participant Feedback

At the end of the formative evaluation, all the participants were asked to complete the embedded feedback form to gather additional information on the changes made and the usefulness of the updated website.

Only 3/40 (8%) participants completed the form. These participants regarded the website and well-being intervention as easy to navigate and useful, as selected from the 5-point Likert scale drop-down menu. They reported that they were “slightly successful” at their selected lifestyle change modules using the 5-point Likert scale drop-down menu. Of these 3 participants, 2 (67%) most enjoyed the “track your progress” function, and 1 (33%) most enjoyed the “wellbeing module.” No comments were made regarding the adaptations implemented.

## Discussion

### Principal Findings

This study adapted an existing health and well-being program to a new audience, namely young persons, using a participatory, co-design approach. Young persons’ mental health is a critical concern following the COVID-19 pandemic, and resources that address the issue at scale are in demand. Furthermore, the use of participatory approaches to develop digital resources for health and well-being is recommended in established guidelines for digital health interventions [62], and useful guidance and examples have been provided [47] to guide the application of these approaches in a health care context with children and young people.

Overall, the study aim was successfully met. A total of 40 young persons were recruited and contributed to a 3-staged participatory co-design study, which resulted in many updates and adaptations made directly by the participants via web-based collaborative group workshops. This was followed by a small formative evaluation of the updated website and well-being intervention.

Each study objective was met. Objectives 1 to 3 were successfully met in stage 1. A total of 24 young persons were consulted and actively engaged in group discussions in which they shared their views and opinions, initial thoughts on the website, and ideas for updates. The participants identified specific areas of the website and well-being intervention that they felt needed to be updated to suit their well-being needs and design preferences. They were also consulted on ways to encourage sustained engagement with the website. They identified ideas that worked for them and considered the content and design features that they felt would enable them to keep going with their selected behavior changes and well-being development.

In stage 2, objective 4 was met. The web-based workshops worked well to provide participants with an opportunity to collaborate with their peers to co-design new content for the website that would be representative of their experiences and would be readable and appropriate for themselves and their peers. The participants engaged in friendly and open discussions with each other guided by a facilitator. The small bite-sized tasks provided the participants with an opportunity to work on and change small areas of the website, one section at a time. This also ensured that the outputs were focused and immediately available for review and discussion. The participants were guided to create the outputs in the workshop as a group. The workshops also included a built-in opportunity to reflect and discuss after the tasks were completed. The small group sizes ensured that each participant could contribute. The outputs were reviewed in successive workshops for data validation and to continue the rapid prototyping. This ensured that all the participants had the opportunity to contribute to multiple website sections. The website name itself was changed from “Champions for Health” to “ACTivate your wellbeing” by the participants, as were several scenarios within the well-being intervention.

The format of the web-based workshops and the use of 2 types of tasks (informed by the participants’ discussions) enabled the opportunity to talk about, draw, and decide on a variety of layouts and design features. For example, the track your progress feature was changed, as were many of the website images, including the home page icons for each of the 5 lifestyle behavior modules and the well-being intervention and key images used within each module. The user dashboard was also changed to incorporate a new display, “streak” function, and new color scheme to coincide with the participants’ ideas about engagement.

Finally, once the updates had been programmed into the website (objective 6), a formative evaluation was undertaken by 40 participants, which led to additional insights into user preferences, likelihood of engagement, and use patterns. Thus, objective 7 was met.

### Comparison With Literature

Winsall et al [63] undertook an exploratory qualitative inquiry to conceptualize how young Australians understood well-being. The authors used workshops to discuss the participants’ understanding of well-being and reported 7 overarching thematic outputs, which identified the multidimensional nature of well-being. In a similar vein, this study explored participants’ views of well-being to adapt an existing digital resource. This approach provided rich qualitative data, which led to the development of design tasks, which were included in a series of participatory co-design workshops, where the participants actively included their understanding, experiences, and conceptualization of well-being in the program directly. In line with the recommendations by Winsall et al [63], activities identified by the target audience as promoting well-being were incorporated directly into the intervention.

Furthermore, Winsall et al [63] reported that participants of different ages had different conceptualizations of well-being. In this study, as the younger cohort engaged more with the program, it is possible that this could explain the difference.

Finally, Winsall et al [63] reported “that young people generally only think about their well-being in times of stress.” This may also lend understanding to the current findings. The initial resource was developed with prevention in mind, as public sector National Health Service staff desired a resource available in times of positive health to build knowledge and understanding to be drawn upon in times of poor mental health. Thus, the initial premise of the intervention and overall program may have hindered adaptation to this new audience. However, resources designed with prevention in mind are critical in the battle against declining mental health, and this issue should be addressed more explicitly in the future.

The formative evaluation (stage 3) saw limited engagement and adherence in half of the participants. However, other studies have reported positive findings. For example, Ponzo et al [64] reported improvements in self-reported anxiety and psychological well-being following a 4-week digital intervention for students, which included mindful components and self-compassion alongside biofeedback and psychoeducation based on cognitive behavioral therapy. Their randomized controlled study included an app and a wearable device, something lacking from this study.

Suffoletto et al [65] explored engagement with an automated digital mental health tool for young persons diagnosed with mental health conditions in America as they transitioned from college to university. The feasibility study reported positive effects on the rates of depression and positive engagement with the tool, which adopted a SMS text message–based empowerment and education approach, in the intervention group compared with the control group; however, no significant differences were found between the 2 groups. The interactive nature of the intervention may explain the difference in engagement, as the study duration was 3 months and participants had higher engagement over the longer duration compared with this study. However, this study differed from our study, as no lifestyle behavior component was included.

A systematic review of 21 digital health interventions for children and young persons [66] provided support for the short-term clinical benefits of this delivery format. Variable use and engagement were highlighted and reiterated debate on the topic poor engagement and adherence as ongoing issues with digital interventions.

Martin et al [67] reported the findings of a co-design process of developing a mobile health intervention called “PEGASO Fit for Future (F4F)” for adolescents from different cultural backgrounds. The authors included 74 participants in a 1 year, 3-staged project. Similar to this study, the participants engaged in the co-design, refinement, and feasibility testing of the new digital program, which also focused on promoting positive lifestyle patterns with a focus on healthy weight. The participants attended a single workshop and were presented with mock-ups or early version prototypes of different apps for user requirements assessment and review. This is in line with the current approach, where the existing website was presented and used to stimulate discussion. In addition, the duration of the formative evaluation in their study was similar to one of the duration options provided in our study, 3 weeks. They piloted the program for 1 and then 2 weeks. The findings of their study are not dissimilar to that of ours. For example, their participants requested personalization, age-appropriate language and content, easy-to-use tutorials, and a reward system. These were all resonated in this study, which sought to personalize the resource for the new age group. For example, current participants identified that the profile area could include the option to upload a profile picture (this was implemented).

Maher et al [68] reported insights from a multifaceted co-design process that was used to explore the topic of engagement with stakeholders within a health care context in New Zealand. Multiple exploratory phases were reported, which enabled the discussion of the topic. Their study highlighted the usefulness of adopting a staged PD process with multiple stakeholders. This study could have benefited from the inclusion of computer programmers and designers at stage 2 to complement and enhance the design processes followed.

Interestingly, most participants who self-selected their duration had the least engagement. It is possible that the initial interest and commitment expressed by those who could self-select their study duration were not activated by the resources, or it is possible that these users selected longer durations to account for their limited free time or reduced ability to prioritize personal self-care. Medical students have different and additional stressors in their academic environment compared with other students [69], which may also explain some of the differences between these 2 student groups. As reported elsewhere in the literature, the use of resources that promote positive well-being is less likely to be undertaken in times of difficulty, as abstract future gains are less motivating [70-72]. For example, the ability to care for one’s own needs during times of distress is often reduced, and patients’ needs are often prioritized by health care professionals [73]. The intervention and the program as a whole could be considered an act of self-care. The practice of self-care varies from person to person. Self-care is defined as an “ability to promote health, prevent disease, maintain health, and cope with illness and disability with or without the support of a health-care provider” [74]; that is, it is the practice of actively looking after one’s own personal mental, physical, and emotional well-being [74].

### Study Limitations

There are several limitations to be considered when reviewing the findings. The study was limited by its small sample size, which limits the generalizability of the findings. However, other studies reported similar sample sizes [75-77].

At each stage, most participants were female. This gender bias limits the application of the findings to males in the target audience demographic. However, as reported by Scapaticci et al [29], female students’ needs are greater, so resources that appeal to females are important.

Not all updates and adaptations identified by the participants during stages 1 and 2 could be incorporated into the website owing to time and cost considerations. In addition, the existing structure of the website meant that some changes could not be implemented. Thus, facilitators must be prepared to explain and outline the limitations or boundaries imposed on participants and the scope available in “bringing to life their creations.” However, it is also important not to restrict the creativity and insights that might arise during workshop sessions.

The division of the participants between the self-selected study duration and the 3-week study duration was not randomized. The programming of the website was such that the groups had to be predefined before registration, and this had to be based on the information provided in the registration form. It was decided that the study program would be used to determine the groups. However, as the website process was anonymous, the researchers did not know who had been allocated to which group and did not interact with the participants during stage 3. Everything was remotely performed. After 12 weeks, the data were extracted and analyzed. Obviously, a control group or randomization procedure would be desirable in future work in this area; however, as randomization was an exploratory element of the study and not the main objective, it was not prioritized. Of note, feasibility trials (this was not a feasibility trial) do not require randomization [78].

Stages 1 and 2 were conducted on the web, as this study was undertaken during the COVID-19 pandemic, and in-person meetings were prohibited in the United Kingdom. This may have limited or altered the manner in which the FG discussions and co-design workshops were facilitated. A discussion of the methodological differences and strengths and limitations of web-based facilitation is warranted in the academic literature, as many studies moved to web-based facilitation, including web-based data collection, from 2020 to 2022; in fact, may continue to do so after advancements in technology and familiarity with web-based software has increased exponentially.

An evaluation of the co-design process itself was not undertaken. Evaluation from the participants’ perspective is advocated [79] to understand the nature of the design and development processes and support future use and application of the methodology outside traditional computer-based fields of research.

Finally, only 3 participants provided feedback following program engagement, which reduced the opportunity to evaluate the intervention and identify the reasons for nonuse or low engagement. Poor engagement and adherence to digital health interventions remains a major issue [80,81], and ways to address this issue should be embedded in research studies.

### Implications and Future Directions

Participants had low baseline well-being, just below the reported cutoff points for CMD. This is of concern, as prior findings highlight the connection between poor student mental health and well-being and low academic achievement and dropout [82-84].

A more streamlined and reduced version of the program could be developed for users who have a similar profile to the GEMs studying a condensed course (these students complete their medical degree in 4 years as opposed to the students enrolled in traditional undergraduate medical degree program, who complete their program in 5 years), as these students face substantial academic pressures to perform and the need for self-care and mental health support has increased exponentially among those in the medical profession in the aftermath of the pandemic [4,73,85].

Alternatively, in line with the suggestions provided by Winsall et al [63], discrete well-being categories could be used to direct and support young persons’ well-being and encourage engagement. Different profile areas could be activated for different students, and tailored interactive features and functionality might make the program more appealing for busy students who are short on time. Technology-based personalized and tailored reminder strategies can support engagement with digital health interventions [86].

Moreover, future research should seek to embed the evaluation of the co-design process into the development phases so that participants’ understanding can be explored and reflected on to support future uses of this process. This is especially in light of the recent National Institute for Health and Care Excellence guidelines [48], which advocate the inclusion of end users in the development of digital and mobile health interventions. The guidance (1.1.8 and 1.1.9) specifies the inclusion of diverse stakeholders throughout all design phases and the need to seek continued feedback. Thus, future studies should seek to embed the evaluation of PD methods and outcomes to ensure that the approaches used are effective and useful.

### Conclusions and Contributions

This study describes the use of a participatory co-design method to redesign an existing health and well-being program for a new audience, namely young persons. Recommendations from the existing literature were followed, and insights from this study were shared for those who wish to use co-design methods with young persons in the context of health and well-being.

The participants actively engaged with the 3-staged participatory process and cocreated new content, selected new images, and discussed layout and presentational and design features. They openly talked about the tools and features that worked for them in other websites and apps that could be incorporated to encourage engagement and adherence, and they were interested in the topic of well-being. The participants who were studying at the university level were pleased to see resources such as the well-being intervention being offered to them and their peers freely as an additional source of support alongside more formal mental health rescues. They wanted to be part of the project to co-design the resources such that they suit their preferences, and the co-design workshops were enjoyable and friendly. They also produced outputs that were then directly inputted into the website, some immediately and some after consultation with external website programmers.

Furthermore, the formative evaluation (stage 3) suggested a potential positive impact from the use of the website, most notably for the users who engaged more with the website, as measured by increased user points. They saw an increase in well-being, as measured by the WEMWBS 3 weeks after the intervention. The comparison between participant groups suggested that a younger audience and shorter intervention duration were associated with higher adherence and completion rates.

Finally, the co-design process was effective. The website now includes images, content, and well-being scenarios that young persons themselves created.

## Appendix

Multimedia Appendix 1 Example resource card task 1a.

## Abbreviations

AAQ-II: Acceptance and Action Questionnaire version 2
ACT: acceptance and commitment therapy
CMD: common mental disorder
FG: focus group
GEM: graduate entry medical student
PD: participatory design
PHQ-4: Patient Health Questionnaire-4
WEMWBS: Warwick Edinburgh Mental Well-Being Scale

## References

[1] (2015). Well-being of future generations (Wales) act 2015. Goverment of United Kingdom.

[2] (2017). Prosperity for all: economic action plan. Llywodraeth Cymru, Government for Wales.

[3] (2021). Written statement: health and social care in Wales – COVID-19: looking forward. Llywodraeth Cymru, Government for Wales.

[4] Cabarkapa S, Nadjidai SE, Murgier J, Ng CH (2020). The psychological impact of COVID-19 and other viral epidemics on frontline healthcare workers and ways to address it: a rapid systematic review. Brain Behav Immun Health.

[5] Chopra S, Ranjan P, Singh V, Kumar S, Arora M, Hasan MS, Kasiraj R, Kaur D, Vikram NK, Malhotra A, Kumari A, Klanidhi KB, Baitha U, Suryansh (2020). Impact of COVID-19 on lifestyle-related behaviours- a cross-sectional audit of responses from nine hundred and ninety-five participants from India. Diabetes Metab Syndr.

[6] Shuja KH, Aqeel M, Jaffar A, Ahmed A (2020). COVID-19 pandemic and impending global mental health implications. Psychiatr Danub.

[7] Hisham IN, Townsend G, Gillard S, Debnath B, Sin J (2021). COVID-19: the perfect vector for a mental health epidemic. BJPsych Bull.

[8] Shevlin M, McBride O, Murphy J, Miller JG, Hartman TK, Levita L, Mason L, Martinez AP, McKay R, Stocks TV, Bennett KM, Hyland P, Karatzias T, Bentall RP (2020). Anxiety, depression, traumatic stress and COVID-19-related anxiety in the UK general population during the COVID-19 pandemic. BJPsych Open.

[9] White RG, Van Der Boor C (2020). Impact of the COVID-19 pandemic and initial period of lockdown on the mental health and well-being of adults in the UK. BJPsych Open.

[10] Dunton GF, Do B, Wang SD (2020). Early effects of the COVID-19 pandemic on physical activity and sedentary behavior in children living in the U.S. BMC Public Health.

[11] Fore HH, Dongyu Q, Beasley DM, Ghebreyesus TA (2020). Child malnutrition and COVID-19: the time to act is now. Lancet.

[12] Galluccio A, Caparello G, Avolio E, Manes E, Ferraro S, Giordano C, Sisci D, Bonofiglio D (2021). Self-perceived physical activity and adherence to the mediterranean diet in healthy adolescents during COVID-19: findings from the DIMENU pilot study. Healthcare (Basel).

[13] Rawat D, Dixit V, Gulati S, Gulati S, Gulati A (2021). Impact of COVID-19 outbreak on lifestyle behaviour: a review of studies published in India. Diabetes Metab Syndr.

[14] (2022). Noncommunicable diseases. World Health Organization.

[15] Brown M, Hooper N, James P, Scott D, Bodger O, John A (2020). A web-delivered acceptance and commitment therapy intervention with email reminders to enhance subjective well-being and encourage engagement with lifestyle behavior change in health care staff: randomized cluster feasibility stud. JMIR Form Res.

[16] Brown M, Hooper N, Eslambolchilar P, John A (2020). Development of a web-based acceptance and commitment therapy intervention to support lifestyle behavior change and well-being in health care staff: participatory design study. JMIR Form Res.

[17] Hubble S, Bolton P (2020). Support for students with mental health issues in higher education in England. UK Parliament.

[18] Sivertsen B, Hysing M, Knapstad M, Harvey AG, Reneflot A, Lønning KJ, O'Connor RC (2019). Suicide attempts and non-suicidal self-harm among university students: prevalence study. BJPsych Open.

[19] Dodd RH, Dadaczynski K, Okan O, McCaffery KJ, Pickles K (2021). Psychological wellbeing and academic experience of university students in Australia during COVID-19. Int J Environ Res Public Health.

[20] Evans S, Alkan E, Bhangoo JK, Tenenbaum H, Ng-Knight T (2021). Effects of the COVID-19 lockdown on mental health, wellbeing, sleep, and alcohol use in a UK student sample. Psychiatry Res.

[21] New J (2017). Colleges struggle to provide ongoing treatment as demands for mental health services increases. Inside Higher Ed.

[22] (2020). Coronavirus and higher education students: England, 20 November to 25 November 2020. Office for National Statistics.

[23] Chen T, Lucock M (2022). The mental health of university students during the COVID-19 pandemic: an online survey in the UK. PLoS One.

[24] Burns D, Dagnall N, Holt M (2020). Assessing the impact of the COVID-19 pandemic on student wellbeing at universities in the United Kingdom: a conceptual analysis. Front Educ.

[25] Thorley C (2017). Not by degrees: improving student mental health in the UK’s universities. Institute for Public Policy Research.

[26] Martinez A, Nguyen S (2020). The impact of covid-19 on college student well-being. Healthy Minds Network & American College Health Association.

[27] (2021). Mental health of children and young people in England 2021 - wave 2 follow up to the 2017 survey. National Health Service Digital.

[28] Graupensperger S, Calhoun BH, Patrick ME, Lee CM (2022). Longitudinal effects of COVID-19-related stressors on young adults' mental health and wellbeing. Appl Psychol Health Well Being.

[29] Scapaticci S, Neri CR, Marseglia GL, Staiano A, Chiarelli F, Verduci E (2022). The impact of the COVID-19 pandemic on lifestyle behaviors in children and adolescents: an international overview. Ital J Pediatr.

[30] Giuntella O, Hyde K, Saccardo S, Sadoff S (2021). Lifestyle and mental health disruptions during COVID-19. Proc Natl Acad Sci U S A.

[31] Seetan K, Al-Zubi M, Rubbai Y, Athamneh M, Khamees A, Radaideh T (2021). Impact of COVID-19 on medical students' mental wellbeing in Jordan. PLoS One.

[32] Sprake EF, Russell JM, Cecil JE, Cooper RJ, Grabowski P, Pourshahidi LK, Barker ME (2018). Dietary patterns of university students in the UK: a cross-sectional study. Nutr J.

[33] Kaess M, Moessner M, Koenig J, Lustig S, Bonnet S, Becker K, Eschenbeck H, Rummel-Kluge C, Thomasius R, Bauer S, ProHEAD Consortium (2021). Editorial perspective: a plea for the sustained implementation of digital interventions for young people with mental health problems in the light of the COVID-19 pandemic. J Child Psychol Psychiatry.

[34] Becker MH (1974). The health belief model and personal health behavior. Health Educ Monogr.

[35] Ajzen I, Fishbein M (1970). The prediction of behavior from attitudinal and normative variables. J Exp Soc Psychol.

[36] Coury J, Schneider JL, Rivelli JS, Petrik AF, Seibel E, D'Agostini B, Taplin SH, Green BB, Coronado GD (2017). Applying the Plan-Do-Study-Act (PDSA) approach to a large pragmatic study involving safety net clinics. BMC Health Serv Res.

[37] Leventhal H, Benyamini Y, Brownlee S, Diefenbach M, Leventhal EA, Patrick-Miller L, Robitaille C, Petrie KJ, Weinman JA (1997). Illness representations: theoretical foundations. Perceptions of Health and Illness: Current Research and Applications.

[38] Hayes SC (2004). Acceptance and commitment therapy, relational frame theory, and the third wave of behavioral and cognitive therapies. Behav Ther.

[39] Ruiz FJ (2012). Acceptance and commitment therapy versus traditional cognitive behavioral therapy: a systematic review and meta-analysis of current empirical evidence. Int J Psychol Psychol Ther.

[40] Barnes-Holmes Y, Hayes SC, Barnes-Holmes D, Roche B (2001). Relational frame theory: a post-Skinnerian account of human language and cognition. Adv Child Dev Behav.

[41] Brown M, Glendenning A, Hoon AE, John A (2016). Effectiveness of web-delivered acceptance and commitment therapy in relation to mental health and well-being: a systematic review and meta-analysis. J Med Internet Res.

[42] Fiorillo D, McLean C, Pistorello J, Hayes SC, Follette VM (2017). Evaluation of a web-based acceptance and commitment therapy program for women with trauma-related problems: a pilot study. J Contextual Behav Sci.

[43] Kishita N, Gould RL, Farquhar M, Contreras M, Van Hout E, Losada A, Cabrera I, Hornberger M, Richmond E, McCracken LM (2022). Internet-delivered guided self-help acceptance and commitment therapy for family carers of people with dementia (iACT4CARERS): a feasibility study. Aging Ment Health.

[44] Lappalainen R, Lappalainen P, Puolakanaho A, Hirvonen R, Eklund K, Ahonen T, Muotka J, Kiuru N (2021). The youth compass - the effectiveness of an online acceptance and commitment therapy program to promote adolescent mental health: a randomized controlled trial. J Contextual Behav Sci.

[45] Larsson A, Hartley S, McHugh L (2022). A randomised controlled trial of brief web-based acceptance and commitment therapy on the general mental health, depression, anxiety and stress of college students. J Contextual Behav Sci.

[46] Pots WT, Fledderus M, Meulenbeek PA, ten Klooster PM, Schreurs KM, Bohlmeijer ET (2016). Acceptance and commitment therapy as a web-based intervention for depressive symptoms: randomised controlled trial. Br J Psychiatry.

[47] Thabrew H, Fleming T, Hetrick S, Merry S (2018). Co-design of eHealth interventions with children and young people. Front Psychiatry.

[48] Cargo M, Mercer SL (2008). The value and challenges of participatory research: strengthening its practice. Annu Rev Public Health.

[49] Jagosh J, Macaulay AC, Pluye P, Salsberg J, Bush PL, Henderson J, Sirett E, Wong G, Cargo M, Herbert CP, Seifer SD, Green LW, Greenhalgh T (2012). Uncovering the benefits of participatory research: implications of a realist review for health research and practice. Milbank Q.

[50] Caixeta MC, Bross JC, Fabricio MM, Tzortzopoulos P (2013). Value generation through user involvement in healthcare design. Proceedings of the 21st Annual Conference of the International Group for Lean Construction.

[51] Palmer VJ, Weavell W, Callander R, Piper D, Richard L, Maher L, Boyd H, Herrman H, Furler J, Gunn J, Iedema R, Robert G (2019). The Participatory Zeitgeist: an explanatory theoretical model of change in an era of coproduction and codesign in healthcare improvement. Med Humanit.

[52] Orlowski SK, Lawn S, Venning A, Winsall M, Jones GM, Wyld K, Damarell RA, Antezana G, Schrader G, Smith D, Collin P, Bidargaddi N (2015). Participatory research as one piece of the puzzle: a systematic review of consumer involvement in design of technology-based youth mental health and well-being interventions. JMIR Hum Factors.

[53] Sanders EB, Brandt E, Binder T (2010). A framework for organizing the tools and techniques of participatory design. Proceedings of the 11th Biennial Participatory Design Conference.

[54] Maheswaran H, Weich S, Powell J, Stewart-Brown S (2012). Evaluating the responsiveness of the Warwick Edinburgh Mental Well-Being Scale (WEMWBS): group and individual level analysis. Health Qual Life Outcomes.

[55] Tennant R, Hiller L, Fishwick R, Platt S, Joseph S, Weich S, Parkinson J, Secker J, Stewart-Brown S (2007). The Warwick-Edinburgh Mental Well-being Scale (WEMWBS): development and UK validation. Health Qual Life Outcomes.

[56] Löwe B, Wahl I, Rose M, Spitzer C, Glaesmer H, Wingenfeld K, Schneider A, Brähler E (2010). A 4-item measure of depression and anxiety: validation and standardization of the Patient Health Questionnaire-4 (PHQ-4) in the general population. J Affect Disord.

[57] Bond FW, Hayes SC, Baer RA, Carpenter KM, Guenole N, Orcutt HK, Waltz T, Zettle RD (2011). Preliminary psychometric properties of the Acceptance and Action Questionnaire-II: a revised measure of psychological inflexibility and experiential avoidance. Behav Ther.

[58] Fledderus M, Bohlmeijer ET, Pieterse ME, Schreurs KM (2012). Acceptance and commitment therapy as guided self-help for psychological distress and positive mental health: a randomized controlled trial. Psychol Med.

[59] Bennett R, Oliver J (2019). Acceptance and Commitment Therapy: 100 Key Points and Techniques.

[60] Braun V, Clarke V (2006). Using thematic analysis in psychology. Qual Res Psychol.

[61] Braun V, Clarke V (2019). Reflecting on reflexive thematic analysis. Qual Res Sport Exerc Health.

[62] (2020). Behaviour change: digital and mobile health interventions. National Institute for Health and Care Excellence.

[63] Winsall M, Orlowski S, Vogl G, Blake V, Nicholas M, Antezana G, Schrader G, Bidargaddi N (2019). Designing online interventions in consideration of young people's concepts of well-being: exploratory qualitative study. JMIR Hum Factors.

[64] Ponzo S, Morelli D, Kawadler JM, Hemmings NR, Bird G, Plans D (2020). Efficacy of the digital therapeutic mobile app BioBase to reduce stress and improve mental well-being among university students: randomized controlled trial. JMIR Mhealth Uhealth.

[65] Suffoletto B, Goldstein T, Gotkiewicz D, Gotkiewicz E, George B, Brent D (2021). Acceptability, engagement, and effects of a mobile digital intervention to support mental health for young adults transitioning to college: pilot randomized controlled trial. JMIR Form Res.

[66] Hollis C, Falconer CJ, Martin JL, Whittington C, Stockton S, Glazebrook C, Davies EB (2017). Annual research review: digital health interventions for children and young people with mental health problems - a systematic and meta-review. J Child Psychol Psychiatry.

[67] Martin A, Caon M, Adorni F, Andreoni G, Ascolese A, Atkinson S, Bul K, Carrion C, Castell C, Ciociola V, Condon L, Espallargues M, Hanley J, Jesuthasan N, Lafortuna CL, Lang A, Prinelli F, Puidomenech Puig E, Tabozzi SA, McKinstry B (2020). A mobile phone intervention to improve obesity-related health behaviors of adolescents across Europe: iterative co-design and feasibility study. JMIR Mhealth Uhealth.

[68] Maher LM, Hayward B, Hayward P, Walsh C (2017). Increasing patient engagement in healthcare service design: a qualitative evaluation of a co-design programme in New Zealand. Patient Exp J.

[69] Levin ME, Hayes SC, Pistorello J, Seeley JR (2016). Web-based self-help for preventing mental health problems in universities: comparing acceptance and commitment training to mental health education. J Clin Psychol.

[70] Cohen D, Winstanley SJ, Greene G (2016). Understanding doctors' attitudes towards self-disclosure of mental ill health. Occup Med (Lond).

[71] Riegel B, Dunbar SB, Fitzsimons D, Freedland KE, Lee CS, Middleton S, Stromberg A, Vellone E, Webber DE, Jaarsma T (2021). Self-care research: where are we now? Where are we going?. Int J Nurs Stud.

[72] Stults-Kolehmainen MA, Sinha R (2014). The effects of stress on physical activity and exercise. Sports Med.

[73] Unadkat S, Farquhar M (2020). Doctors' wellbeing: self-care during the COVID-19 pandemic. BMJ.

[74] (2020). Self care during COVID-19. World Health Organization.

[75] Elf M, Rystedt H, Lundin J, Krevers B (2012). Young carers as co-designers of a web-based support system: the views of two publics. Inform Health Soc Care.

[76] Kelders SM, Pots WT, Oskam MJ, Bohlmeijer ET, van Gemert-Pijnen JE (2013). Development of a web-based intervention for the indicated prevention of depression. BMC Med Inform Decis Mak.

[77] Wadley G, Lederman R, Gleeson J, Alvarez-Jimenez M (2013). Participatory design of an online therapy for youth mental health. Proceedings of the 25th Australian Computer-Human Interaction Conference: Augmentation, Application, Innovation, Collaboration.

[78] Arain M, Campbell MJ, Cooper CL, Lancaster GA (2010). What is a pilot or feasibility study? A review of current practice and editorial policy. BMC Med Res Methodol.

[79] Bossen C, Dindler C, Iversen OS (2016). Evaluation in participatory design: a literature survey. Proceedings of the 14th Participatory Design Conference: Full papers - Volume 1.

[80] Bubolz S, Mayer G, Gronewold N, Hilbel T, Schultz JH (2020). Adherence to established treatment guidelines among unguided digital interventions for depression: quality evaluation of 28 web-based programs and mobile apps. J Med Internet Res.

[81] Linardon J, Fuller-Tyszkiewicz M (2020). Attrition and adherence in smartphone-delivered interventions for mental health problems: a systematic and meta-analytic review. J Consult Clin Psychol.

[82] Eisenberg D, Golberstein E, Hunt JB (2009). Mental health and academic success in college. BE J Econ Anal Policy.

[83] Hysenbegasi A, Hass SL, Rowland CR (2005). The impact of depression on the academic productivity of university students. J Ment Health Policy Econ.

[84] Neale I, Piggott L, Fagence S, Unite Students, YouGov, Youthsight (2016). Student resilience: unite students insight report 2016. Academic Cooperation Association.

[85] Maben J, Bridges J (2020). COVID-19: supporting nurses' psychological and mental health. J Clin Nurs.

[86] Khwaja M, Pieritz S, Faisal AA, Matic A (2021). Personality and engagement with digital mental health interventions. Proceedings of the 29th ACM Conference on User Modeling, Adaptation and Personalization.

