# Supplementary material for: Adaptation of ACTivate Your Wellbeing, a Digital Health and Well-being Program for Young Persons: Co-design Approach
Source: JMIR Form Res. 2023 Apr 13;7:e39913. doi: 10.2196/39913 (PMC10141270; doi:10.2196/39913)
Supplement: Multimedia Appendix 1 [file formative_v7i1e39913_app1.docx]

# Multimedia Appendix 1. Table S1. Example resource card task 1a.

#### Task 1a: Re-design the Home page

In this task you are asked to consider ways in which to redesign the home page so that it is displayed as you / your peers would like to see it.

Feedback indicated that the placement of different components of the home page were not entirely helpful for promoting use and understanding of the website.

1. Use the images provided of the home page as it is now (with the different components: header text and banner image / 6 modules / 123 steps / testimonials / bottom banner and logos) below to guide you.
2. Discuss it together and then map out how you might want it to look. You can do this on word, on paper or however you wish. Reorder the images shown below or re design them.
3. Consider the following;
   - The website has been renamed ACTIVATE your well-being. Are you happy with this? If not what should / could it be?
   - The header text has been updated. Are you happy with this? What could/should it say here?
   - the fruit bowl image received mixed opinions. Search unsplash for a new image(s) and select one to be used. Email the options you found and the one you chose to me.
   - What order should the lifestyle modules be displayed in?
   - Select a new image for each of the lifestyle modules. Search unsplash for a new image(s)
   - Rewrite the introduction text under each lifestyle module so that it appeals to you / your peers.
   - Identify and add in any additional text to explain the purpose of the website to support you / your peers understanding and use of the website.
   - If you use any other websites as reference or inspiration please keep a note of this and email it to me.
   - Consider main menu and any additional menu items you think would be helpful.


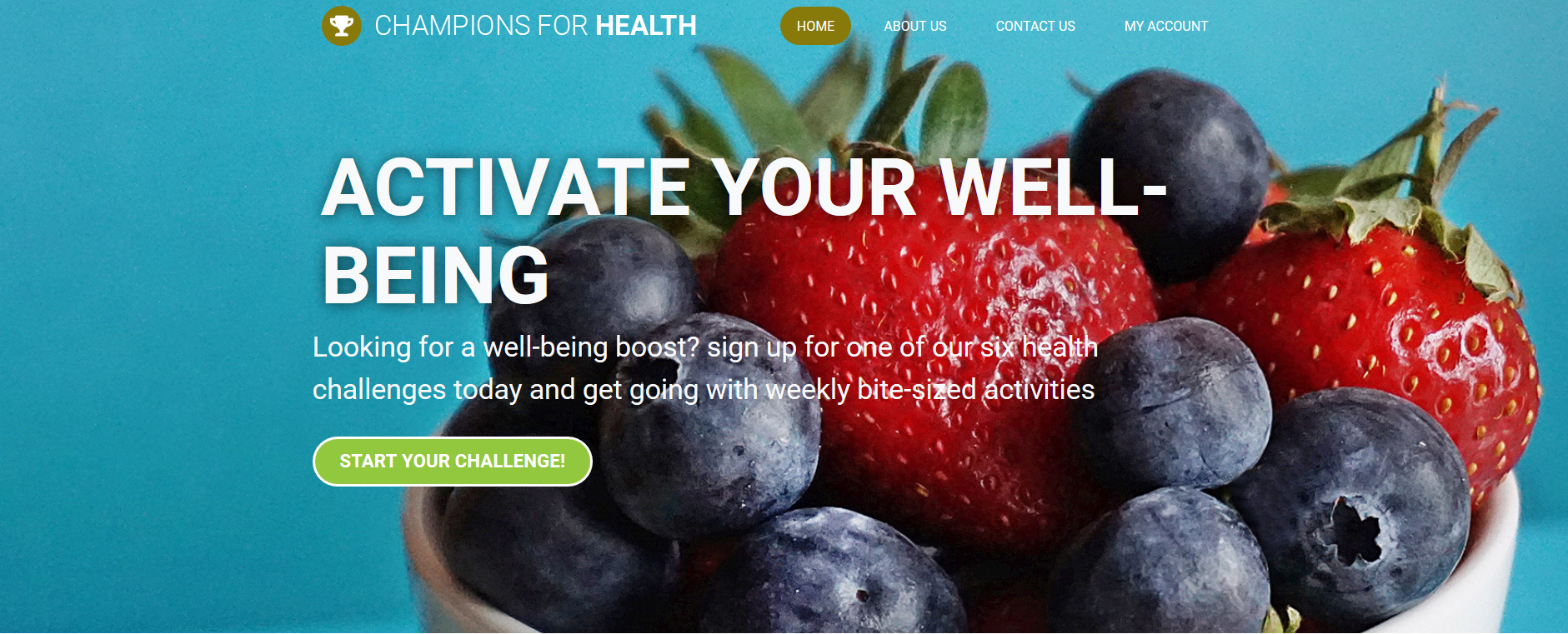


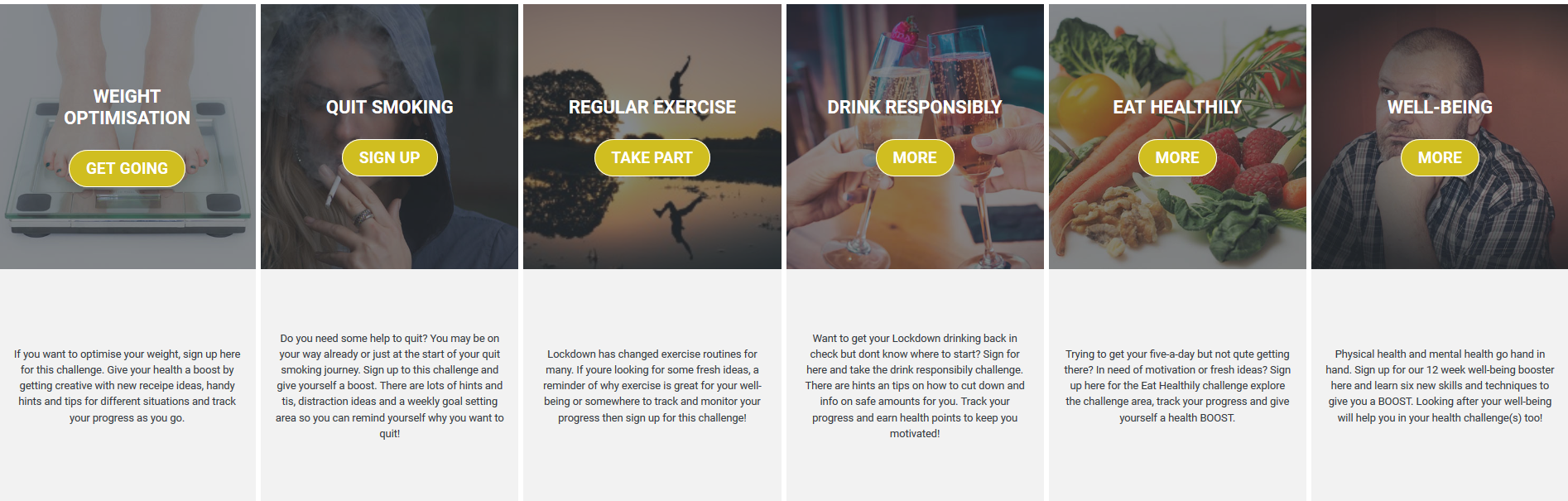


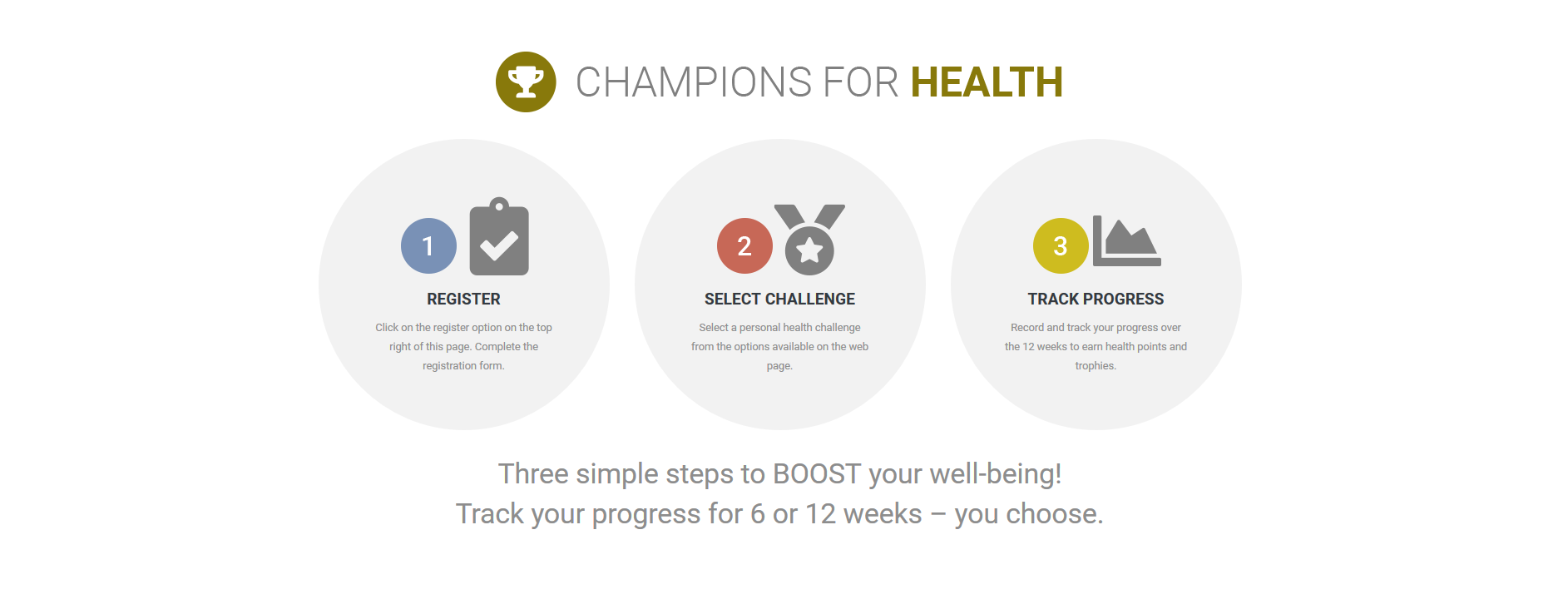


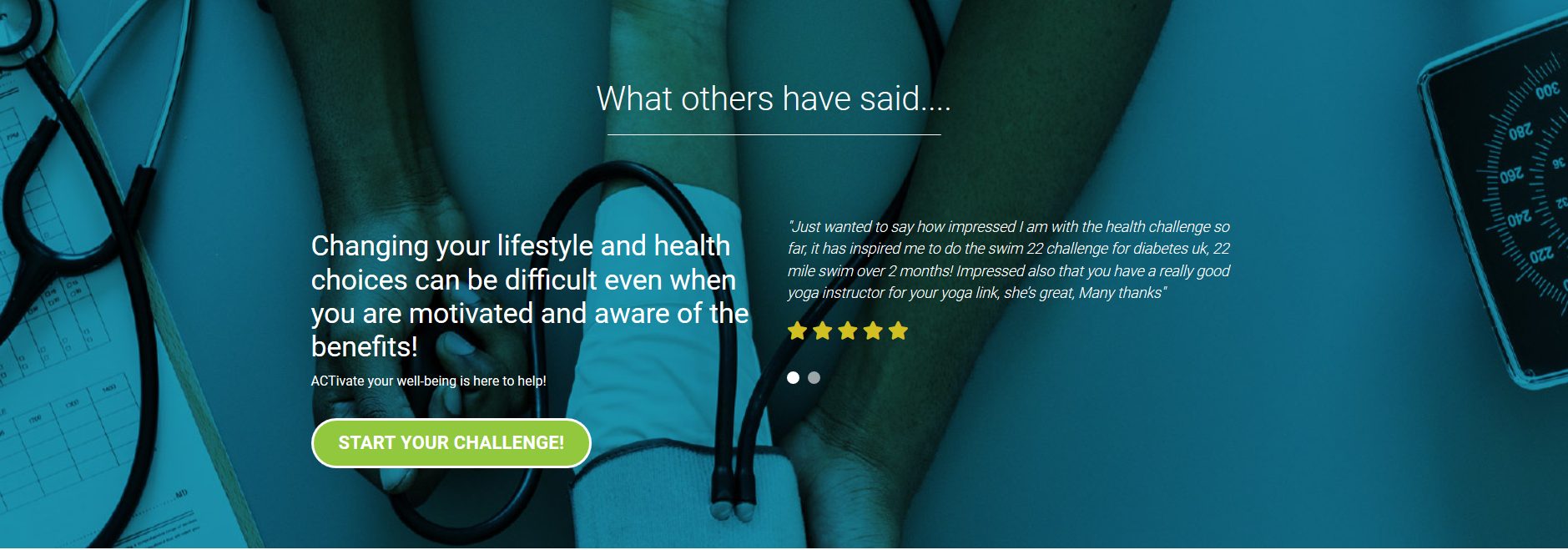


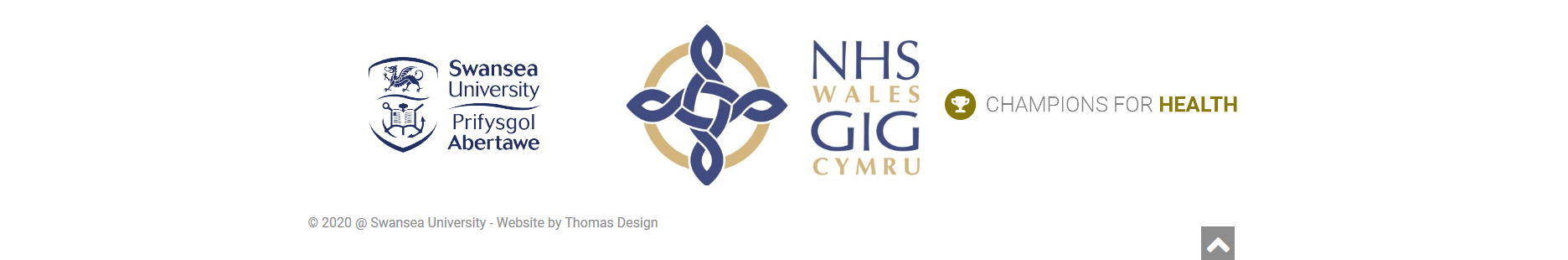


**Extracts from you / your peers feedback questionnaires which may guide you in your task**

| Navigation | 3 steps at the bottom of the page could be included in what you first see, as this gives a bit more information into what Champions for health really is about |
| --- | --- |
|  | I think the 3 steps could be closer to the top or be the first thing you would see on the website. |
| Navigation | The three circles explaining how to use the product should be on a separate “how to” page |
| Navigation | the arrangement of the [lifestyle] modules could be tweaked so they are arranged in a more ‘neutral’ way. Maybe alphabetically. Just because I think that weight and smoking are big things that a lot more people struggle with; and seeing those two modules first might be a bit off putting. |
| Visual appeal  Navigation | I think the “welcome” text is too large and this also pushes all the links so far down the page that it forces people to scroll before they are visible. |
| Visual appeal  Engagement | To better engage the viewer, could use more dynamic images or images with more vibrant colours to represent each health challenge. |
|  | I think the pictures are all great for the challenges, but I think it would be nice to have a picture of a person or a group of people at the top of the page, to give a more personal feel to the website |
| Content update  Engagement | More testimonials would definitely be needed as only two, with one being one sentence, aren’t going to encourage anyone to take the programme up. |
|  | I think the main page could benefit from something such as imagery or quotes that could add to motivate people as soon as they log in and see the front page. I think motivation is a significant component of succeeding in any challenge and motivating the website “user/customer” could be beneficial. |
| Content update  Engagement | I think having a bit more outline of what the plans actually involve may entice more people to click and read further. |
| Content update | I’d expect to see links to social media sites etc on the front page. |
